# Supplementary material for: Therapeutic outcomes with surgical and medical management for primary aldosteronism: protocol for a systematic review and meta-analysis
Source: BMJ Open. 2023 Jul 30;13(7):e072585. doi: 10.1136/bmjopen-2023-072585 (PMC10387625; doi:10.1136/bmjopen-2023-072585)
Supplement: Supplementary data [file bmjopen-2023-072585supp001.pdf]

Supplementary material. Full search strategies used in PubMed, Web of Science, and EMBASE to identify relevant articles for the systematic review.

PubMed

#	Searches
#1	hyperaldosteronism[tiab] OR hyperaldosteronemia[tiab] OR aldosteronism[tiab] OR aldosteronemia[tiab] OR Hyperaldosteronism[mh] OR Conn Syndrome[tiab] OR Primary Hyperaldosteronism[tiab] OR Conn's Syndrome[tiab] OR Conns Syndrome[tiab] OR Syndrome, Conn*[tiab]
#2	Adrenalectomy[mh] OR adrenalectomy[tiab] OR adrenalectomies[tiab] OR surgery[tiab] OR surgical[tiab] OR resection[tiab] OR management[tiab] OR treatment[tiab] OR drug therapy[mh] OR medical therapy[tiab] OR MR antagonists[tiab] OR MR antagonist[tiab] OR mineralocorticoid receptor antagonists[tiab] OR mineralocorticoid receptor antagonist[tiab] OR Spironolactone[mh] OR spironolactone[tiab] OR eplerenone[mh] OR eplerenone[tiab] OR Spirolactone[tiab] OR Veroshpiron[tiab] OR Verospirone[tiab] OR Spiractin[tiab] OR Spirogamma[tiab] OR Spirolang[tiab] OR Aldactone[tiab] OR Verospiron[tiab] OR Aldactone A[tiab] OR Aquareduct[tiab] OR Espironolactona Alter[tiab] OR SC-9420[tiab] OR SC 9420[tiab] OR SC9420[tiab] OR Inspra[tiab]
#3	#1 AND #2
#4	Animals[mh] NOT humans[mh]
#5	#3 NOT #4
#6	Limit to year 2000-

Web of Science Core Collection

#	Searches
#1	TS=(hyperaldosteronism OR hyperaldosteronemia OR aldosteronism OR aldosteronemia OR Hyperaldosteronism OR "Conn Syndrome" OR "Primary Hyperaldosteronism" OR "Conn's Syndrome" OR "Conns Syndrome" OR "Syndrome, Conn*")
#2	TS=(Adrenalectomy OR adrenalectomies OR surgery OR surgical OR resection OR management OR treatment OR "drug therapy" OR "medical therapy" OR ”MR antagonist” OR "MR antagonists" OR ”mineralocorticoid receptor antagonists” OR ”mineralocorticoid receptor antagonist” OR Spironolactone OR spironolactone OR eplerenone OR eplerenone OR Spirolactone OR Veroshpiron OR Verospirone OR Spiractin OR Spirogamma OR Spirolang OR Aldactone OR Verospiron OR "Aldactone A" OR Aquareduct OR "Espironolactona Alter" OR SC-9420 OR "SC 9420" OR SC9420 OR Inspra)
#3	#1 AND #2
#4	ALL=human*
#5	#3 NOT #4
#6	Limit to year 2000-, Articles

Embase

#	Searches
#1	hyperaldosteronism:ti,ab OR hyperaldosteronemia:ti,ab OR aldosteronism:ti,ab OR aldosteronemia:ti,ab OR Hyperaldosteronism/exp OR 'Conn Syndrome':ti,ab OR 'Primary Hyperaldosteronism':ti,ab OR 'Conns Syndrome':ti,ab OR 'Syndrome, Conn*':ti,ab
#2	Adrenalectomy/exp OR adrenalectomy:ti,ab OR adrenalectomies:ti,ab OR surgery/exp OR surgery:ti,ab OR surgical:ti,ab OR resection:ti,ab OR mangament/exp OR management:ti,ab OR treatment:ti,ab OR 'drug therapy'/exp OR 'medical therapy':ti,ab OR 'MR antagonists':ti,ab OR ’MR antagonist’:ti,ab OR ’mineralocorticoid receptor antagonists’:ti,ab OR ’mineralocorticoid receptor antagonist’:ti,ab OR Spironolactone/exp OR spironolactone:ti,ab OR eplerenone/exp OR eplerenone:ti,ab OR Spirolactone:ti,ab OR Veroshpiron:ti,ab OR Verospirone:ti,ab OR Spiractin:ti,ab OR Spirogamma:ti,ab OR Spirolang:ti,ab OR Aldactone:ti,ab OR Verospiron:ti,ab OR 'Aldactone A':ti,ab OR Aquareduct:ti,ab OR 'Espironolactona Alter':ti,ab OR SC-9420:ti,ab OR 'SC 9420':ti,ab OR SC9420:ti,ab OR Inspra:ti,ab
#3	#1 AND #2
#4	('animal'/exp OR animal:ti,ab) NOT (human/exp OR human:ti,ab)
#5	#3 NOT #4
#6	Limit to year 2000-, Articles
