# Supplementary material for: Therapeutic outcomes with surgical and medical management for primary aldosteronism: protocol for a systematic review and meta-analysis
Source: BMJ Open. 2023 Jul 30;13(7):e072585. doi: 10.1136/bmjopen-2023-072585 (PMC10387625; doi:10.1136/bmjopen-2023-072585)
Supplement: Author's manuscript [file bmjopen-2023-072585.draft_revisions.pdf]

BMJ Open is committed to open peer review. As part of this commitment we make the peer review history of every article we publish publicly available.

When an article is published we post the peer reviewers' comments and the authors' responses online. We also post the versions of the paper that were used during peer review. These are the versions that the peer review comments apply to.

The versions of the paper that follow are the versions that were submitted during the peer review process. They are not the versions of record or the final published versions. They should not be cited or distributed as the published version of this manuscript.

BMJ Open is an open access journal and the full, final, typeset and author-corrected version of record of the manuscript is available on our site with no access controls, subscription charges or pay-per-view fees (http://bmjopen.bmj.com).

If you have any questions on BMJ Open's open peer review process please email info.bmjopen@bmj.com

BMJ Open

Therapeutic Outcomes with Surgical and Medical Management for Primary Aldosteronism: protocol for a Systematic Review and Meta-Analysis

Journal:	*BMJ Open*
Manuscript ID	bmjopen-2023-072585
Article Type:	Protocol
Date Submitted by the Author:	11-Feb-2023
Complete List of Authors:	Rocca, Aldo; University of Molise<br>Gkaniatsa, Eleftheria; University of Gothenburg, Department of Internal Medicine and Clinical Nutrition; Sahlgrenska University Hospital, Department of Endocrinology<br>Brunese, Maria Chiara; University of Molise, Department of Medicine and Health Science “V. Tiberio”<br>Hessman, Eva; University of Gothenburg<br>Muth, Andreas; Sahlgrenska University Hospital, Department of Surgery; University of Gothenburg, Department of Surgery<br>Nwaru, Bright; University of Gothenburg, Krefting Research Centre<br>Ragnarsson, Oskar; Sahlgrenska University Hospital, Department of Medicine<br>Bobbio, Emanuele; Sahlgrenska University Hospital, Department of Cardiology; University of Gothenburg<br>Esposito, Daniela; University of Gothenburg, Department of Internal Medicine and Clinical Nutrition; Sahlgrenska University Hospital, Department of Endocrinology
Keywords:	Hypertension < CARDIOLOGY, General endocrinology < DIABETES & ENDOCRINOLOGY, Endocrine tumours < DIABETES & ENDOCRINOLOGY, Adrenal disorders < DIABETES & ENDOCRINOLOGY

SCHOLARONE™
Manuscripts

Therapeutic Outcomes with Surgical and Medical Management for Primary Aldosteronism: protocol for a Systematic Review and Meta-Analysis

Aldo Rocca[1], MD, Eleftheria Gkaniatsa[2,3], MD, Maria Chiara Brunese[1], MD, Eva Hessman[4], Andreas Muth[5,6], MD, PhD, Bright I. Nwaru[7,8], PhD, Oskar Ragnarsson[2,3,8], MD, PhD, Emanuele Bobbio[9,10]*, MD, Daniela Esposito[2,3]*, MD, PhD

*these authors contributed equally

[1] *Department of Medicine and Health Science "V. Tiberio", University of Molise, Campobasso, Italy*
[2] *Department of Internal Medicine and Clinical Nutrition, Institute of Medicine, Sahlgrenska Academy, University of Gothenburg, Gothenburg, Sweden*
[3] *Department of Endocrinology, Sahlgrenska University Hospital, Gothenburg, Sweden*
[4] *Biomedical Library, Gothenburg University Library, University of Gothenburg, Gothenburg, Sweden*
[5] *Department of Surgery, Sahlgrenska University Hospital, Gothenburg, Sweden*
[6] *Department of Surgery, Institute of Clinical Sciences, Sahlgrenska Academy, University of Gothenburg, Gothenburg, Sweden*
[7] *Krefting Research Centre, Institute of Medicine, Sahlgrenska Academy, University of Gothenburg, Gothenburg, Sweden*
[8] *Wallenberg Centre for Molecular and Translational Medicine, Institute of Medicine, University of Gothenburg, Gothenburg, Sweden*
[9] *Department of Cardiology, Sahlgrenska University Hospital, Gothenburg, Sweden*
[10] *Institute of Medicine at Sahlgrenska Academy, University of Gothenburg, Gothenburg, Sweden*

Key Words: hyperaldosteronism, adrenalectomy, MR antagonists, spironolactone, eplerenone, meta-analysis

Abstract: 257 words

Manuscript: 1756 words

References: 29

Tables and figures: 1

Running title: Therapeutic outcomes in primary aldosteronism

For peer review only

Corresponding author and person to whom reprint requests should be addressed:

Daniela Esposito, MD, PhD, Department of Endocrinology, Sahlgrenska University Hospital Institute of Medicine, Sahlgrenska Academy, University of Gothenburg, Gothenburg, Sweden.
Email: daniela.esposito@gu.se

For peer review only

E-mail address of all protocol authors:

Aldo Rocca: aldo.rocca@unimol.it

Eleftheria Gkaniatsa: eleftheria.gkaniatsa@vgregion.se

Maria Chiara Brunese: mariachiarabrunese@gmail.com

Eva Hessman: eva.hessman@ub.gu.se

Andreas Muth: andreas.muth@vgregion.se

Bright I. Nwaru: bright.nwaru@gu.se

Oskar Ragnarsson: oskar.ragnarsson@medic.gu.se

Emanuele Bobbio: emanuele.bobbio@vgregion.se

Daniela Esposito: daniela.esposito@gu.se

For peer review only

Abstract

Introduction: Treatment strategies for primary aldosteronism (PA) include unilateral adrenalectomy and medical treatment with mineralocorticoid receptor (MR) antagonists. Whether these two different treatment strategies are comparable in mitigating the detrimental effect of PA on outcomes is still debated.

Objectives: The primary aim of this systematic review is to identify, appraise, and synthesize existing literature comparing clinical outcomes after treatment in patients with PA.

Methods and analysis: A systematic and comprehensive search will be performed using PubMed, Google scholar, Web of Science, and EMBASE, for studies published until December 2022. Observational and interventional studies will be eligible for inclusion. The quality of observational studies will be assessed using the Newcastle–Ottawa scale, while interventional studies will be assessed using the Cochrane Effective Practice Organization of Care tool. The collected evidence will be narratively synthesized. We will perform meta-analysis to pool estimates from studies considered to be homogenous. Reporting of the systematic review and meta-analysis will be in accordance to the Meta-analysis of Observational Studies in Epidemiology Preferred Reporting Items for Systematic reviews and Meta-Analysis guidelines.

Ethics and dissemination: As this study is based solely on the published literature, no ethics approval is required. This review will aim to provide some estimates on outcomes, including survival, rates of clinical and biochemical control, cardiovascular and cerebrovascular events, as well as data on quality of life and renal function, in patients with PA treated surgically or with MR antagonists. The study findings will be presented at scientific meetings and will be published in an international peer-reviewed scientific journal.

PROSPERO registration number: CRD42022362506.

For peer review only

Strengths and limitations of this study

- Findings from this systematic review will provide a comprehensive and most contemporaneous synthesis of the underlying evidence regarding long-term outcomes in patients with primary aldosteronism (PA) who have been treated with either surgery or mineralocorticoid receptor (MR) antagonists.
- This systematic review protocol follows the Preferred Reporting Items for Systematic Review and Meta-Analysis Protocols guidelines.
- The identification of studies from leading medical and public health databases, with no geographical or language limitations, will advance the import of this evidence synthesis across settings.
- The reproducibility of our work is enhanced through a priori outline of the review processes before the actual review starts.

For peer review only

For peer review only - http://bmjopen.bmj.com/site/about/guidelines.xhtml

1. Background

Primary aldosteronism (PA), also known as Conn´s syndrome, is the most common cause of secondary hypertension, affecting 3–13% of hypertensive patients and 20% of patients with therapy-resistant hypertension (1). PA is characterized by autonomous hypersecretion of aldosterone, causing inappropriate potassium excretion, concurrent sodium and fluid retention, as well as low plasma renin concentration (1, 2). PA is most commonly caused by adrenal adenoma or unilateral or bilateral adrenal hyperplasia (2). Rare causes of PA include adrenocortical carcinoma and inherited forms of familial hyperaldosteronism (1, 2). Patients with PA are at higher risk of cardiovascular diseases such as myocardial infarction, stroke and atrial fibrillation than patients with essential hypertension also when adjusted for blood pressure (BP) (3, 4).

Treatment strategies for PA include unilateral adrenalectomy and medical treatment with mineralocorticoid receptor (MR) antagonists (1). Minimally invasive adrenalectomy is a safe and feasible procedure and recommended treatment for patients with unilateral PA (5-7). Although adrenalectomy usually results in biochemical cure, hypertension can persist in up to 60% of patients (7). Conversely, MR antagonists (spironolactone or eplerenone) are an option in patients with bilateral PA or when surgery is not feasible (8). Furthermore, medical treatment is preferred in case of uncertain lateralization, especially at centers where adrenal venous sampling is not available or has been unsuccessful (9).

Evidence is still limited on whether different treatment strategies mitigate the detrimental effect of PA on short- and long-term outcomes (10). Some investigators reported similar outcomes between surgical and medical treatment (11-13). However, other studies showed better control of arterial BP and hypokalemia, better cardiovascular outcomes, as well as improved quality of life after adrenalectomy than with medical treatment (5). In a recent meta-analysis, lower risk of all-cause mortality and/or major adverse cardiovascular events (MACE) were also reported in surgically treated patients compared with those treated with MR antagonists (14). However, evidence about the effect of adrenalectomy *vs* MR antagonists on clinical and/or biochemical control as well as quality of life and renal function is still debated. Furthermore, evaluation of effects and side effects of MR antagonist treatment is hampered by patients frequently being undertreated. Thus, it is paramount to identify and synthesize the available evidence to date in order to gain clearer insights into this field.

For peer review only - http://bmjopen.bmj.com/site/about/guidelines.xhtml

2. Scientific questions and objectives

The aim of this systematic review is to identify, critically appraise, and synthesize available data evaluating whether patients with PA who underwent adrenalectomy have higher survival rate than patients treated with MR antagonists.

The secondary aim of this review is to compare rates of cardiovascular and cerebrovascular events (heart failure, myocardial infarction, stroke), successful clinical control (normalized or improved blood pressure, tapering of antihypertensive medications), biochemical control (normalized plasma renin and potassium levels), as well as improved quality of life and renal function between patients who underwent adrenalectomy and those treated with MR antagonists.

3. Project description

3.1 Methods

We will perform a systematic review and meta-analysis according to Preferred Reporting Items for Systematic Reviews and Meta-Analysis (PRISMA) guidelines (15).

The main study objectives have been formulated according to PICO (Patients, Intervention, Comparison, Outcomes) model (**Table 1**). The protocol for this review has been registered in PROSPERO (registration number: CRD42022362506).

For peer review only - http://bmjopen.bmj.com/site/about/guidelines.xhtml

Table 1. PICO

**P**	Patients with PA
**I**	Adrenalectomy
**C**	Medical treatment
**O**	*All-cause mortality*<br>*Cardiovascular and cerebrovascular events:* heart failure, myocardial infarction, stroke<br>*Clinical control:* normalized or improved blood pressure, less antihypertensive medication<br>*Biochemical control:* normalized renin and potassium<br>*Quality of life*<br>*Renal function*

3.2 Eligibility criteria and information sources

The population of interest will be patients with PA treated with adrenalectomy in comparison with those receiving MR antagonists. Studies that report survival rates and rates of clinical control (i.e., normalized or improved blood pressure, tapering of antihypertensive medication), biochemical control (i.e. normalized renin and potassium), cardiovascular and cerebrovascular events (heart failure, myocardial infarction, stroke), as well as data on quality of life or renal function will be included.

Data across different age groups, gender, and ethnicity will be analyzed, and there will be no exclusion by country or language. We will translate studies reported in other languages than English.

Observational studies (cross-sectional, cohort, and case–control) and interventional (randomized controlled trials, field trials, community trials, etc.) studies will be included.

Exclusion criteria will be: date of publication before 2000, review articles, case reports, editorials, letters, and other articles where the calculation of rates of outcomes is not feasible. If an eligible article does not report the rates of the pre-specified outcomes, an attempt to retrieve this data will be performed by contacting the study authors.

For peer review only

For peer review only - http://bmjopen.bmj.com/site/about/guidelines.xhtml

Search will be conducted in four databases (PubMed, Google scholar, Web of Science, and EMBASE) using commonly used terms stated in related literature as well as Medical Subject Headings (MeSH) terms. Search strategies will be focused on three concepts: the population/diagnosis, surgical treatment and medical therapy. The following terms will be used: hyperaldosteronism, hyperaldosteronaemia, aldosteronism, aldosteronaemia, adrenalectomy(-ies), surgery, surgical resection, MR antagonists, spironolactone, eplerenone.

All databases will be searched from January 2000 until December 2022. An additional manual-search based on the citations and references for included reports will also be conducted. All reports obtained from the databases will be exported to the reference management software Endnote. The search result will then be deduplicated, following the Bramer method (16).

3.3 Ethical issues

Ethical approval or informed consent are not required since this systematic review will be based only on previously published studies and does not imply any direct contact with individual subjects.

3.4 Selection process

Article screening will be independently performed by authors AR, EG, MB and DE by evaluating titles and abstracts for eligibility assessment. Relevant reports will be identified and analyzed by at least two authors in a blinded process using the web application Rayyan (17). In case of disagreement, a third author will resolve it. Bibliographies and citations for the eligible articles will be examined to identify any additional relevant report, which were not retrieved in the literature search.

The selected reports will be categorized into three groups: irrelevant, relevant, and unsure. The reports categorized as irrelevant by both reviewers will be excluded. The reports categorized as relevant or unsure by at least one reviewer will be retrieved and the full papers evaluated. Then, for all the potentially eligible reports, the full texts will be carefully reviewed and a selection of reports to include will be made by each reviewer. The reports selected by the reviewers will be compared and any discrepancies will be discussed. When an agreement is not reached, another author (OR) will make the final decision.

The search and selection process will be presented in a PRISMA flowchart.

For peer review only

3.5 Data extraction

Relevant information from included studies will be extracted using a data extraction form that will help to standardize the data extraction process. The following data will be collected: study name, name of the first author, year of publication, country where the study was conducted, total number of patients, source from which study subjects were selected, study design, outcomes definition, gender, age, ethnicity, incidence with 95% confidence intervals (CIs) of the outcomes. When possible, missing information will be calculated from the available data. Authors will be contacted by the reviewers if data are inadequate, unclear, or missing from the report. At least two reviewers will independently extract data from all studies. Any discrepancies between reviewers in the data extraction will be resolved by either consensus or a third reviewer will arbitrate.

For peer review only

3.6 Quality assessment

Quality of observational studies will be assessed using the Newcastle – Ottawa Scale (NOS), while the Cochrane Effective Practice Organization of Care tool will be used for interventional studies. Two reviewers will perform the quality appraisal and a third reviewer will arbitrate any disagreement.

3.7 Data synthesis

If possible, relative Risk (RR) estimates together with Confidence Intervals (CI) will be calculated from each study and, if the studies are sufficiently homogeneous, a meta-analysis will be performed by pooling estimates across studies using the random effects approach. Heterogeneity will be examined using the Higgins I^2 statistic (18). Heterogeneity will be considered minimal if I^2 values will be between 0-30%, moderate if I^2 will be between 31-50%, and high if I^2 >50%.

Publication bias will be assessed using Begg's funnel plot and Egger's test. An asymmetrical funnel plot or P value < 0.10 on Egger's test will be considered as an indication of publication bias. If data are sufficient, subgroup analyses will be performed based on the quality of study, age, gender, disease severity, diagnostic strategy, and ethnicity.

For peer review only - http://bmjopen.bmj.com/site/about/guidelines.xhtml

3.8 Patient and Public Involvement

None

4. Discussion

Once thought a rare disease, PA is nowadays known to be the most frequent form of secondary hypertension, affecting 3–13% of hypertensive patients (1, 19). PA is associated with higher risk of metabolic, renal, and cardiovascular complications compared with essential hypertension (20). The current treatment options for PA includes surgical adrenalectomy or medical therapy with MR antagonists and the choice of treatment is based on PA subtype classifications and patient preference (14, 21, 22). In small clinical studies, surgical treatment has been shown to have better outcomes (9, 10, 19). However, large, randomized trials are lacking, and no solid conclusion can be drawn.

Previous meta-analyses performed on this topic resulted in controversial findings, probably due to a high degree of heterogeneity between the included studies and small sample sizes (5, 23). Also, the comparison between surgical and medical treatment in PA is challenging. Surgical approach has changed over time, with introduction of minimally invasive surgery and robotic approaches that have modified the panorama of the PA treatment, allowing for organ-sparing resection and improved surgical outcomes (24, 25). Therefore, studies published before 2000 will be excluded in the present meta-analysis. Another possible pitfall could be that patients receiving MR antagonists are often undertreated, resulting in an inadequate biochemical and clinical control of the disease (6, 26). In undertreated patients, the plasma renin remains suppressed, which *per se* results in increased risk of cardiovascular events, renal dysfunction, and mortality (6). On the other hand, adrenalectomy solves the root cause of PA and, thus, the MR over-activation, making the comparison with medical treatment inaccurate (6).

Hence, it is inaccurate to compare this group of patients to those treated with adrenalectomy that solves the root cause of PA and may result in better outcomes (27-29).

Still, no previous study has synthesized the available evidence to date about the effect of different treatment strategies on clinical and/or biochemical control as well as quality of life

For peer review only - http://bmjopen.bmj.com/site/about/guidelines.xhtml

and renal function. In the present review, we will investigate long-term outcomes in patients with PA who have been treated with either surgery or MR antagonists. In addition, we will gather information on demographic characteristics (age, gender, and ethnicity/race) in order to explore their potential impact on treatment outcomes. Overall, we believe that our systemic review will provide valuable information for optimizing treatment strategies in patients with PA.

Acknowledgements

We thank medical librarians Mrs. Eva Hessman and Mrs. Linda Hammarbäck at the Biomedical Library, Gothenburg University Library, University of Gothenburg, Gothenburg, Sweden for performing the systematical search in the literature and for their valuable feedback on our search strategy and methodology.

Contributions

OR and DE conceived this study. AR, EG, MCB, EB and DE developed the study protocol and will implement the systematic review under the supervision of AM, BN, and OR. EB and BN will provide the statistical analysis expertise. EB, BN, and DE will conduct data analysis. EH will develop the search strings and conduct the study search. AR, EG, MCB, and DE will take part in the study search, and will perform the screening and extraction of data whereas AM, BN, OR, and EB will review the work. DE wrote the first manuscript draft and all authors gave input to the final draft of the protocol.

Funding statement

This work received support from the Swedish state under the agreement between the Swedish government and the county councils, the ALF-agreement (ALFGBG-874631).

Conflicts of interest and financial disclosure

For peer review only

None declared

For peer review only

References

1. Young WF. Primary aldosteronism: renaissance of a syndrome. Clin Endocrinol (Oxf). 2007;66(5):607-18.

2. Conn JW, Louis LH. Primary aldosteronism: a new clinical entity. Trans Assoc Am Physicians. 1955;68:215-31; discussion, 31-3.

3. Monticone S, D'Ascenzo F, Moretti C, Williams TA, Veglio F, Gaita F, et al. Cardiovascular events and target organ damage in primary aldosteronism compared with essential hypertension: a systematic review and meta-analysis. Lancet Diabetes Endocrinol. 2018;6(1):41-50.

4. Catena C, Colussi G, Nadalini E, Chiuch A, Baroselli S, Lapenna R, et al. Cardiovascular outcomes in patients with primary aldosteronism after treatment. Arch Intern Med. 2008;168(1):80-5.

5. Muth A, Ragnarsson O, Johannsson G, Wangberg B. Systematic review of surgery and outcomes in patients with primary aldosteronism. Br J Surg. 2015;102(4):307-17.

6. Funder JW, Carey RM, Mantero F, Murad MH, Reincke M, Shibata H, et al. The Management of Primary Aldosteronism: Case Detection, Diagnosis, and Treatment: An Endocrine Society Clinical Practice Guideline. J Clin Endocrinol Metab. 2016;101(5):1889-916.

7. Iacobone M, Citton M, Viel G, Rossi GP, Nitti D. Approach to the surgical management of primary aldosteronism. Gland Surg. 2015;4(1):69-81.

8. Stavropoulos K, Papadopoulos C, Koutsampasopoulos K, Lales G, Mitas C, Doumas M. Mineralocorticoid Receptor Antagonists in Primary Aldosteronism. Curr Pharm Des. 2018;24(46):5508-16.

9. Williams TA, Burrello J, Sechi LA, Fardella CE, Matrozova J, Adolf C, et al. Computed Tomography and Adrenal Venous Sampling in the Diagnosis of Unilateral Primary Aldosteronism. Hypertension. 2018;72(3):641-9.

10. Park KS, Kim JH, Yang YS, Hong AR, Lee DH, Moon MK, et al. Outcomes analysis of surgical and medical treatments for patients with primary aldosteronism. Endocr J. 2017;64(6):623-32.

For peer review only

11. Kline GA, Pasieka JL, Harvey A, So B, Dias VC. Medical or surgical therapy for primary aldosteronism: post-treatment follow-up as a surrogate measure of comparative outcomes. Ann Surg Oncol. 2013;20(7):2274-8.

12. Wachtel H, Fraker DL. Therapeutic Outcomes with Surgical and Medical Management of Primary Aldosteronism. Curr Cardiol Rep. 2021;23(7):89.

13. Sechi LA, Colussi GL, Novello M, Uzzau A, Catena C. Mineralocorticoid Receptor Antagonists and Clinical Outcomes in Primary Aldosteronism: As Good as Surgery? Horm Metab Res. 2015;47(13):1000-6.

14. Chen SY, Chen JY, Huang WC, Puar THK, Chin Kek P, Chueh JS, et al. Cardiovascular outcomes and all-cause mortality in primary aldosteronism after adrenalectomy or mineralocorticoid receptor antagonist treatment: a meta-analysis. Eur J Endocrinol. 2022;187(6):S47-S58.

15. Page MJ, McKenzie JE, Bossuyt PM, Boutron I, Hoffmann TC, Mulrow CD, et al. The PRISMA 2020 statement: an updated guideline for reporting systematic reviews. Bmj. 2021;372:n71.

16. Bramer WM, Giustini D, de Jonge GB, Holland L, Bekhuis T. De-duplication of database search results for systematic reviews in EndNote. J Med Libr Assoc. 2016;104(3):240-3.

17. Ouzzani M, Hammady H, Fedorowicz Z, Elmagarmid A. Rayyan-a web and mobile app for systematic reviews. Syst Rev. 2016;5(1):210.

18. Higgins JP, Thompson SG. Quantifying heterogeneity in a meta-analysis. Stat Med. 2002;21(11):1539-58.

19. Huang WC, Chen YY, Lin YH, Chueh JS. Composite Cardiovascular Outcomes in Patients With Primary Aldosteronism Undergoing Medical Versus Surgical Treatment: A Meta-Analysis. Front Endocrinol (Lausanne). 2021;12:644260.

20. Rayner B. Primary aldosteronism and aldosterone-associated hypertension. J Clin Pathol. 2008;61(7):825-31.

21. Spyroglou A, Handgriff L, Müller L, Schwarzlmüller P, Parasiliti-Caprino M, Fuss CT, et al. The metabolic phenotype of patients with primary aldosteronism: impact of subtype and sex - a multicenter-study of 3566 Caucasian and Asian subjects. Eur J Endocrinol. 2022;187(3):361-72.

For peer review only

For peer review only - http://bmjopen.bmj.com/site/about/guidelines.xhtml

22. Morimoto S, Ichihara A. Management of primary aldosteronism and mineralocorticoid receptor-associated hypertension. Hypertens Res. 2020;43(8):744-53.

23. Satoh M, Maruhashi T, Yoshida Y, Shibata H. Systematic review of the clinical outcomes of mineralocorticoid receptor antagonist treatment versus adrenalectomy in patients with primary aldosteronism. Hypertens Res. 2019;42(6):817-24.

24. Brunt LM. The positive impact of laparoscopic adrenalectomy on complications of adrenal surgery. Surg Endosc. 2002;16(2):252-7.

25. Colvin J, Krishnamurthy V, Jin J, Shin J, Siperstein A, Berber E. A Comparison of Robotic Versus Laparoscopic Adrenalectomy in Patients With Primary Hyperaldosteronism. Surg Laparosc Endosc Percutan Tech. 2017;27(5):391-3.

26. Hundemer GL, Curhan GC, Yozamp N, Wang M, Vaidya A. Cardiometabolic outcomes and mortality in medically treated primary aldosteronism: a retrospective cohort study. Lancet Diabetes Endocrinol. 2018;6(1):51-9.

27. Reincke M, Fischer E, Gerum S, Merkle K, Schulz S, Pallauf A, et al. Observational study mortality in treated primary aldosteronism: the German Conn's registry. Hypertension. 2012;60(3):618-24.

28. Rossi GP, Cesari M, Cuspidi C, Maiolino G, Cicala MV, Bisogni V, et al. Long-term control of arterial hypertension and regression of left ventricular hypertrophy with treatment of primary aldosteronism. Hypertension. 2013;62(1):62-9.

29. Sechi LA, Novello M, Lapenna R, Baroselli S, Nadalini E, Colussi GL, et al. Long-term renal outcomes in patients with primary aldosteronism. JAMA. 2006;295(22):2638-45.

For peer review only

1

PRISMA-P 2015 Checklist

This checklist has been adapted for use with protocol submissions to *Systematic Reviews* from Table 3 in Moher D et al: Preferred reporting items for systematic review and meta-analysis protocols (PRISMA-P) 2015 statement. *Systematic Reviews* 2015 **4**:1

Section/topic	#	Checklist item	Information reported		Line number(s)
			Yes	No	
**ADMINISTRATIVE INFORMATION**					
**Title**					
Identification	1a	Identify the report as a protocol of a systematic review	X		
Update	1b	If the protocol is for an update of a previous systematic review, identify as such		X	
**Registration**	2	If registered, provide the name of the registry (e.g., PROSPERO) and registration number in the Abstract	X		
**Authors**					
Contact	3a	Provide name, institutional affiliation, and e-mail address of all protocol authors; provide physical mailing address of corresponding author	X		
Contributions	3b	Describe contributions of protocol authors and identify the guarantor of the review	X		
**Amendments**	4	If the protocol represents an amendment of a previously completed or published protocol, identify as such and list changes; otherwise, state plan for documenting important protocol amendments		X	
**Support**					
Sources	5a	Indicate sources of financial or other support for the review	X		
Sponsor	5b	Provide name for the review funder and/or sponsor	X		
Role of sponsor/funder	5c	Describe roles of funder(s), sponsor(s), and/or institution(s), if any, in developing the protocol		X	
**INTRODUCTION**					
**Rationale**	6	Describe the rationale for the review in the context of what is already known	X		
**Objectives**	7	Provide an explicit statement of the question(s) the review will address with reference to participants, interventions, comparators, and outcomes (PICO)	X		
**METHODS**					

For peer review only

BioMed Central The Open Access Publisher

Section/topic	#	Checklist item	Information reported: Yes	Information reported: No	Line number(s)
**Eligibility criteria**	8	Specify the study characteristics (e.g., PICO, study design, setting, time frame) and report characteristics (e.g., years considered, language, publication status) to be used as criteria for eligibility for the review	X		
**Information sources**	9	Describe all intended information sources (e.g., electronic databases, contact with study authors, trial registers, or other grey literature sources) with planned dates of coverage	X		
**Search strategy**	10	Present draft of search strategy to be used for at least one electronic database, including planned limits, such that it could be repeated		X	
STUDY RECORDS					
Data management	11a	Describe the mechanism(s) that will be used to manage records and data throughout the review	X		
Selection process	11b	State the process that will be used for selecting studies (e.g., two independent reviewers) through each phase of the review (i.e., screening, eligibility, and inclusion in meta-analysis)	X		
Data collection process	11c	Describe planned method of extracting data from reports (e.g., piloting forms, done independently, in duplicate), any processes for obtaining and confirming data from investigators	X		
**Data items**	12	List and define all variables for which data will be sought (e.g., PICO items, funding sources), any pre-planned data assumptions and simplifications	X		
**Outcomes and prioritization**	13	List and define all outcomes for which data will be sought, including prioritization of main and additional outcomes, with rationale	X		
**Risk of bias in individual studies**	14	Describe anticipated methods for assessing risk of bias of individual studies, including whether this will be done at the outcome or study level, or both; state how this information will be used in data synthesis	X		
DATA					
**Synthesis**	15a	Describe criteria under which study data will be quantitatively synthesized	X		
	15b	If data are appropriate for quantitative synthesis, describe planned summary measures, methods of handling data, and methods of combining data from studies, including any planned exploration of consistency (e.g., I^2, Kendall's tau)	X		
	15c	Describe any proposed additional analyses (e.g., sensitivity or subgroup analyses, meta-regression)	X		
	15d	If quantitative synthesis is not appropriate, describe the type of summary planned		X	
**Meta-bias(es)**	16	Specify any planned assessment of meta-bias(es) (e.g., publication bias across studies, selective reporting within studies)	X		
**Confidence in cumulative evidence**	17	Describe how the strength of the body of evidence will be assessed (e.g., GRADE)	X		

For peer review only

For peer review only - http://bmjopen.bmj.com/site/about/guidelines.xhtml

BMJ Open

Therapeutic Outcomes with Surgical and Medical Management for Primary Aldosteronism: protocol for a Systematic Review and Meta-Analysis

Journal:	*BMJ Open*
Manuscript ID	bmjopen-2023-072585.R1
Article Type:	Protocol
Date Submitted by the Author:	10-Apr-2023
Complete List of Authors:	Rocca, Aldo; University of Molise, Department of Medicine and Health Science “V. Tiberio”<br>Gkaniatsa, Eleftheria; University of Gothenburg, Department of Internal Medicine and Clinical Nutrition, Institute of Medicine, Sahlgrenska Academy,; Sahlgrenska University Hospital, Department of Endocrinology<br>Brunese, Maria Chiara; University of Molise, Department of Medicine and Health Science “V. Tiberio”<br>Hessman, Eva; Gothenburg University Library, Biomedical Library,<br>Muth, Andreas; Sahlgrenska University Hospital, Department of Surgery, ; University of Gothenburg, Department of Surgery, Institute of Clinical Sciences, Sahlgrenska Academy<br>Nwaru, Bright; University of Gothenburg, Krefting Research Centre; University of Gothenburg, Wallenberg Centre for Molecular and Translational Medicine, Institute of Medicine,<br>Ragnarsson, Oskar; Sahlgrenska University Hospital, Department of Endocrinology; University of Gothenburg, Department of Internal Medicine and Clinical Nutrition, Institute of Medicine, Sahlgrenska Academy<br>Bobbio, Emanuele; Sahlgrenska University Hospital, Department of Cardiology; University of Gothenburg, Institute of Medicine at Sahlgrenska Academy<br>Esposito, Daniela; University of Gothenburg, Department of Internal Medicine and Clinical Nutrition, Institute of Medicine, Sahlgrenska Academy, ; Sahlgrenska University Hospital, Department of Endocrinology
**Primary Subject Heading**:	Diabetes and endocrinology
Secondary Subject Heading:	Cardiovascular medicine
Keywords:	Hypertension < CARDIOLOGY, General endocrinology < DIABETES & ENDOCRINOLOGY, Endocrine tumours < DIABETES & ENDOCRINOLOGY, Adrenal disorders < DIABETES & ENDOCRINOLOGY

SCHOLARONE™
Manuscripts

Therapeutic Outcomes with Surgical and Medical Management for Primary Aldosteronism: protocol for a Systematic Review and Meta-Analysis

Aldo Rocca[1], MD, Eleftheria Gkaniatsa[2,3], MD, Maria Chiara Brunese[1], MD, Eva Hessman[4], Andreas Muth[5,6], MD, PhD, Bright I. Nwaru[7,8], PhD, Oskar Ragnarsson[2,3,8], MD, PhD, Emanuele Bobbio[9,10]*, MD, Daniela Esposito[2,3]*, MD, PhD

*these authors contributed equally

[1] Department of Medicine and Health Science "V. Tiberio", University of Molise, Campobasso, Italy
[2] Department of Internal Medicine and Clinical Nutrition, Institute of Medicine, Sahlgrenska Academy, University of Gothenburg, Gothenburg, Sweden
[3] Department of Endocrinology, Sahlgrenska University Hospital, Gothenburg, Sweden
[4] Biomedical Library, Gothenburg University Library, University of Gothenburg, Gothenburg, Sweden
[5] Department of Surgery, Sahlgrenska University Hospital, Gothenburg, Sweden
[6] Department of Surgery, Institute of Clinical Sciences, Sahlgrenska Academy, University of Gothenburg, Gothenburg, Sweden
[7] Krefting Research Centre, Institute of Medicine, Sahlgrenska Academy, University of Gothenburg, Gothenburg, Sweden
[8] Wallenberg Centre for Molecular and Translational Medicine, Institute of Medicine, University of Gothenburg, Gothenburg, Sweden
[9] Department of Cardiology, Sahlgrenska University Hospital, Gothenburg, Sweden
[10] Institute of Medicine at Sahlgrenska Academy, University of Gothenburg, Gothenburg, Sweden

Key Words: hyperaldosteronism, adrenalectomy, MR antagonists, spironolactone, eplerenone, meta-analysis

Abstract: 257 words

Manuscript: 1945 words

References: 30

Tables and figures: 1

Running title: Therapeutic outcomes in primary aldosteronism

For peer review only

Corresponding author and person to whom reprint requests should be addressed:

Daniela Esposito, MD, PhD, Department of Endocrinology, Sahlgrenska University Hospital Institute of Medicine, Sahlgrenska Academy, University of Gothenburg, Gothenburg, Sweden.
Email: daniela.esposito@gu.se

For peer review only

E-mail address of all protocol authors:

Aldo Rocca: aldo.rocca@unimol.it
Eleftheria Gkaniatsa: eleftheria.gkaniatsa@vgregion.se
Maria Chiara Brunese: mariachiarabrunese@gmail.com
Eva Hessman: eva.hessman@ub.gu.se
Andreas Muth: andreas.muth@vgregion.se
Bright I. Nwaru: bright.nwaru@gu.se
Oskar Ragnarsson: oskar.ragnarsson@medic.gu.se
Emanuele Bobbio: emanuele.bobbio@vgregion.se
Daniela Esposito: daniela.esposito@gu.se

For peer review only

Abstract

Introduction: Treatment strategies for primary aldosteronism (PA) include unilateral adrenalectomy and medical treatment with mineralocorticoid receptor (MR) antagonists. Whether these two different treatment strategies are comparable in mitigating the detrimental effect of PA on outcomes is still debated.

Objectives: The primary aim of this systematic review is to identify, appraise, and synthesize existing literature comparing clinical outcomes after treatment in patients with PA.

Methods and analysis: A systematic and comprehensive search will be performed using PubMed, Google scholar, Web of Science, and EMBASE, for studies published until December 2022. Observational and interventional studies will be eligible for inclusion. The quality of observational studies will be assessed using the Newcastle–Ottawa scale, while interventional studies will be assessed using the Cochrane Effective Practice Organization of Care tool. The collected evidence will be narratively synthesized. We will perform meta-analysis to pool estimates from studies considered to be homogenous. Reporting of the systematic review and meta-analysis will be in accordance to the Meta-analysis of Observational Studies in Epidemiology Preferred Reporting Items for Systematic reviews and Meta-Analysis guidelines.

Ethics and dissemination: As this study is based solely on the published literature, no ethics approval is required. This review will aim to provide some estimates on outcomes, including survival, rates of clinical and biochemical control, cardiovascular and cerebrovascular events, as well as data on quality of life and renal function, in patients with PA treated surgically or with MR antagonists. The study findings will be presented at scientific meetings and will be published in an international peer-reviewed scientific journal.

PROSPERO registration number: CRD42022362506.

For peer review only

Strengths and limitations of this study

- Findings from this systematic review will provide a comprehensive and most contemporaneous synthesis of the underlying evidence regarding long-term outcomes in patients with primary aldosteronism (PA) who have been treated with either surgery or mineralocorticoid receptor (MR) antagonists.
- This systematic review protocol follows the Preferred Reporting Items for Systematic Review and Meta-Analysis Protocols guidelines.
- The identification of studies from leading medical and public health databases, with no geographical or language limitations, will advance the import of this evidence synthesis across settings.
- The reproducibility of our work is enhanced through a priori outline of the review processes before the actual review starts.

For peer review only

1. Background

Primary aldosteronism (PA), also known as Conn´s syndrome, is the most common cause of secondary hypertension, affecting 3–13% of hypertensive patients and 20% of patients with therapy-resistant hypertension (1). PA is characterized by autonomous hypersecretion of aldosterone, causing inappropriate potassium excretion, concurrent sodium and fluid retention, as well as low plasma renin concentration (1, 2). PA is most commonly caused by adrenal adenoma or unilateral or bilateral adrenal hyperplasia (2). Rare causes of PA include adrenocortical carcinoma and inherited forms of familial hyperaldosteronism (1, 2). Patients with PA are at higher risk of cardiovascular diseases such as myocardial infarction, stroke and atrial fibrillation than patients with essential hypertension also when adjusted for blood pressure (BP) (3, 4).

Treatment strategies for PA include unilateral adrenalectomy and medical treatment with mineralocorticoid receptor (MR) antagonists (1). Minimally invasive adrenalectomy is a safe and feasible procedure and recommended treatment for patients with unilateral PA (5-7). Although adrenalectomy usually results in biochemical cure, hypertension can persist in up to 60% of patients (7). Conversely, MR antagonists (spironolactone or eplerenone) are an option in patients with bilateral PA or when surgery is not feasible (8). Furthermore, medical treatment is preferred in case of uncertain lateralization, especially at centers where adrenal venous sampling is not available or has been unsuccessful (9).

Evidence is still limited on whether different treatment strategies mitigate the detrimental effect of PA on short- and long-term outcomes (10). Some investigators reported similar outcomes between surgical and medical treatment (11-13). However, other studies showed better control of arterial BP and hypokalemia, better cardiovascular outcomes, as well as improved quality of life after adrenalectomy than with medical treatment (5). In a recent meta-analysis, lower risk of all-cause mortality and/or major adverse cardiovascular events (MACE) were also reported in surgically treated patients compared with those treated with MR antagonists (14). However, evidence about the effect of adrenalectomy *vs* MR antagonists on clinical and/or biochemical control as well as quality of life and renal function is still debated. Furthermore, evaluation of effects and side effects of MR antagonist treatment is hampered by patients frequently being undertreated. Thus, it is paramount to identify and synthesize the available evidence to date in order to gain clearer insights into this field.

For peer review only - http://bmjopen.bmj.com/site/about/guidelines.xhtml

2. Scientific questions and objectives

The aim of this systematic review is to identify, critically appraise, and synthesize available data evaluating whether patients with PA who underwent adrenalectomy have higher survival rate than patients treated with MR antagonists.

The secondary aim of this review is to compare rates of cardiovascular and cerebrovascular events (heart failure, myocardial infarction, stroke), successful clinical control (normalized or improved blood pressure, tapering of antihypertensive medications), biochemical control (normalized plasma renin and potassium levels), as well as improved quality of life and renal function between patients who underwent adrenalectomy and those treated with MR antagonists.

3. Project description

3.1 Methods

We will perform a systematic review and meta-analysis according to Preferred Reporting Items for Systematic Reviews and Meta-Analysis (PRISMA) guidelines (15).

The main study objectives have been formulated according to PICO (Patients, Intervention, Comparison, Outcomes) model (**Table 1**). The protocol for this review has been performed according PRISMA-P guidelines and has been registered in PROSPERO (registration number: CRD42022362506).

This systematic review was planned in September 2022 and is expected to be finalized in January 2024.

For peer review only

Table 1. PICO

**P**	Patients with PA
**I**	Adrenalectomy
**C**	Medical treatment
**O**	*All-cause mortality*<br>*Cardiovascular and cerebrovascular events:* heart failure, myocardial infarction, stroke<br>*Clinical control:* normalized or improved blood pressure, less antihypertensive medication<br>*Biochemical control:* normalized renin and potassium<br>*Quality of life*<br>*Renal function*

3.2 Eligibility criteria and information sources

The population of interest will be patients with PA treated with adrenalectomy in comparison with those receiving MR antagonists. Studies that report survival rates and rates of clinical control (i.e., normalized or improved blood pressure, tapering of antihypertensive medication), biochemical control (i.e. normalized renin and potassium), cardiovascular and cerebrovascular events (heart failure, myocardial infarction, stroke), as well as data on quality of life or renal function will be included.

Data across different age groups, gender, and ethnicity will be analyzed, and there will be no exclusion by country or language. We will translate studies reported in other languages than English.

Observational studies (cross-sectional, cohort, and case–control) and interventional (randomized controlled trials, field trials, community trials, etc.) studies will be included.

Exclusion criteria will be: date of publication before 2000, review articles, case reports, editorials, letters, and other articles where the calculation of rates of outcomes is not feasible.

For peer review only - http://bmjopen.bmj.com/site/about/guidelines.xhtml

If an eligible article does not report the rates of the pre-specified outcomes, an attempt to retrieve this data will be performed by contacting the study authors.

Search will be conducted in four databases (PubMed, Google scholar, Web of Science, and EMBASE) using commonly used terms stated in related literature as well as Medical Subject Headings (MeSH) terms. Search strategies will be focused on three concepts: the population/diagnosis, surgical treatment and medical therapy. The following terms will be used: hyperaldosteronism, hyperaldosteronaemia, aldosteronism, aldosteronaemia, adrenalectomy(-ies), surgery, surgical resection, MR antagonists, spironolactone, eplerenone.

All databases will be searched from January 2000 until December 2022. An additional manual-search based on the citations and references for included reports will also be conducted. All reports obtained from the databases will be exported to the reference management software Endnote. The search result will then be deduplicated, following the Bramer method (16).

3.3 Ethical issues

Ethical approval or informed consent are not required since this systematic review will be based only on previously published studies and does not imply any direct contact with individual subjects.

3.4 Selection process

Article screening will be independently performed by authors AR, EG, MB and DE by evaluating titles and abstracts for eligibility assessment. Relevant reports will be identified and analyzed by at least two authors in a blinded process using the web application Rayyan (17). In case of disagreement, a third author will resolve it. Bibliographies and citations for the eligible articles will be examined to identify any additional relevant report, which were not retrieved in the literature search.

The selected reports will be categorized into three groups: irrelevant, relevant, and unsure. The reports categorized as irrelevant by both reviewers will be excluded. The reports categorized as relevant or unsure by at least one reviewer will be retrieved and the full papers evaluated. Then, for all the potentially eligible reports, the full texts will be carefully reviewed and a selection of reports to include will be made by each reviewer. The reports selected by the reviewers will be compared and any discrepancies will be discussed. When an agreement is not reached, another author (OR) will make the final decision.

For peer review only - http://bmjopen.bmj.com/site/about/guidelines.xhtml

The search and selection process will be presented in a PRISMA flowchart.

3.5 Data extraction

Relevant information from included studies will be extracted using a data extraction form that will help to standardize the data extraction process. The following data will be collected: study name, name of the first author, year of publication, country where the study was conducted, total number of patients, source from which study subjects were selected, study design, aetiology of PA, definition of diagnostic criteria for PA, outcomes definition, gender, age, ethnicity, incidence with 95% confidence intervals (CIs) of the outcomes. When possible, missing information will be calculated from the available data. Authors will be contacted by the reviewers if data are inadequate, unclear, or missing from the report. At least two reviewers will independently extract data from all studies. Any discrepancies between reviewers in the data extraction will be resolved by either consensus or a third reviewer will arbitrate.

3.6 Quality assessment

Quality of observational studies will be assessed using the Newcastle – Ottawa Scale (NOS), while the Cochrane Effective Practice Organization of Care tool will be used for interventional studies. Two reviewers will perform the quality appraisal and a third reviewer will arbitrate any disagreement.

3.7 Data synthesis

If possible, relative Risk (RR) estimates together with Confidence Intervals (CI) will be calculated from each study and, if the studies are sufficiently homogeneous, a meta-analysis will be performed by pooling estimates across studies using the random effects approach. Heterogeneity will be examined using the Higgins I^2 statistic (18). Heterogeneity will be considered minimal if I^2 values will be between 0-30%, moderate if I^2 will be between 31-50%, and high if I^2 >50%.

Publication bias will be assessed using Begg's funnel plot and Egger's test. An asymmetrical funnel plot or P value < 0.10 on Egger's test will be considered as an indication of publication

For peer review only

bias. If data are sufficient, subgroup analyses will be performed based on the quality of study, age, gender, etiology of PA, disease severity, diagnostic criteria for PA, and ethnicity.

3.8 Patient and Public Involvement

None

4. Discussion

Once thought a rare disease, PA is nowadays known to be the most frequent form of secondary hypertension, affecting 3–13% of hypertensive patients (1, 19). PA is associated with higher risk of metabolic, renal, and cardiovascular complications compared with essential hypertension (20). The current treatment options for PA includes surgical adrenalectomy or medical therapy with MR antagonists and the choice of treatment is based on PA subtype classifications and patient preference (14, 21, 22). In small clinical studies, surgical treatment has been shown to have better outcomes (9, 10, 19). However, large, randomized trials are lacking, and no solid conclusion can be drawn.

Previous systematic reviews and meta-analyses performed on this topic (5, 14, 23, 24) have mainly assessed the impact of adrenalectomy vs medical treatment on all-cause mortality and major adverse cardiovascular events (*e.g.* coronary artery disease, stroke, arrhythmia, and heart failure) in PA patients. However, other treatment outcomes, as clinical and biochemical control, quality of life and other comorbidities (rather than cardiovascular) have not been assessed yet.

The comparison between surgical and medical treatment in PA is challenging. Surgical approach has changed over time, with introduction of minimally invasive surgery and robotic approaches that have modified the panorama of the PA treatment, allowing for organ-sparing resection and improved surgical outcomes (25, 26). Therefore, studies published before 2000 will be excluded in the present meta-analysis. Another possible pitfall could be that patients receiving MR antagonists are often undertreated, resulting in an inadequate biochemical and clinical control of the disease (6, 27). In undertreated patients, the plasma renin remains suppressed, which *per se* results in increased risk of cardiovascular events, renal dysfunction, and mortality (6). On the other hand, adrenalectomy solves the root cause of PA and, thus,

For peer review only - http://bmjopen.bmj.com/site/about/guidelines.xhtml

the MR over-activation, making the comparison with medical treatment challenging (6). It is also important to consider that patients who are treated with surgery may have different disease characteristics compared to those treated medically (*e.g.* unilateral vs. bilateral PA or uncertain lateralization), which may affect treatment outcomes. Additionally, many PA patients may not be suitable candidates for surgery due to various reasons, such as patient preference or comorbidities. These factors can confound the comparison between surgical and medical therapy for PA; therefore the interpretation of the results of this comparison should be done with caution, and individualized treatment decisions should be made (28-30).

Still, no previous study has synthesized the available evidence to date about the effect of different treatment strategies on clinical and/or biochemical control as well as quality of life and renal function. In the present review, we will investigate long-term outcomes in patients with PA who have been treated with either surgery or MR antagonists. In addition, we will gather information on demographic characteristics (age, gender, and ethnicity/race) in order to explore their potential impact on treatment outcomes. Overall, we believe that our systemic review will provide valuable information for optimizing treatment strategies in patients with PA.

Acknowledgements

We thank medical librarians Mrs. Eva Hessman and Mrs. Linda Hammarbäck at the Biomedical Library, Gothenburg University Library, University of Gothenburg, Gothenburg, Sweden for performing the systematical search in the literature and for their valuable feedback on our search strategy and methodology.

Contributions

OR and DE conceived this study. AR, EG, MCB, EB and DE developed the study protocol and will implement the systematic review under the supervision of AM, BN, and OR. EB and BN will provide the statistical analysis expertise. EB, BN, and DE will conduct data analysis. EH will develop the search strings and conduct the study search. AR, EG, MCB, and DE will take part in the study search, and will perform the screening and extraction of data whereas AM,

For peer review only - http://bmjopen.bmj.com/site/about/guidelines.xhtml

BN, OR, and EB will review the work. DE wrote the first manuscript draft and all authors gave input to the final draft of the protocol.

Funding statement

This work received support from the Swedish state under the agreement between the Swedish government and the county councils, the ALF-agreement (ALFGBG-874631).

Conflicts of interest and financial disclosure

None declared

For peer review only

References

1. Young WF. Primary aldosteronism: renaissance of a syndrome. Clin Endocrinol (Oxf). 2007;66(5):607-18.

2. Conn JW, Louis LH. Primary aldosteronism: a new clinical entity. Trans Assoc Am Physicians. 1955;68:215-31; discussion, 31-3.

3. Monticone S, D'Ascenzo F, Moretti C, Williams TA, Veglio F, Gaita F, et al. Cardiovascular events and target organ damage in primary aldosteronism compared with essential hypertension: a systematic review and meta-analysis. Lancet Diabetes Endocrinol. 2018;6(1):41-50.

4. Catena C, Colussi G, Nadalini E, Chiuch A, Baroselli S, Lapenna R, et al. Cardiovascular outcomes in patients with primary aldosteronism after treatment. Arch Intern Med. 2008;168(1):80-5.

5. Muth A, Ragnarsson O, Johannsson G, Wangberg B. Systematic review of surgery and outcomes in patients with primary aldosteronism. Br J Surg. 2015;102(4):307-17.

6. Funder JW, Carey RM, Mantero F, Murad MH, Reincke M, Shibata H, et al. The Management of Primary Aldosteronism: Case Detection, Diagnosis, and Treatment: An Endocrine Society Clinical Practice Guideline. J Clin Endocrinol Metab. 2016;101(5):1889-916.

7. Iacobone M, Citton M, Viel G, Rossi GP, Nitti D. Approach to the surgical management of primary aldosteronism. Gland Surg. 2015;4(1):69-81.

8. Stavropoulos K, Papadopoulos C, Koutsampasopoulos K, Lales G, Mitas C, Doumas M. Mineralocorticoid Receptor Antagonists in Primary Aldosteronism. Curr Pharm Des. 2018;24(46):5508-16.

9. Williams TA, Burrello J, Sechi LA, Fardella CE, Matrozova J, Adolf C, et al. Computed Tomography and Adrenal Venous Sampling in the Diagnosis of Unilateral Primary Aldosteronism. Hypertension. 2018;72(3):641-9.

10. Park KS, Kim JH, Yang YS, Hong AR, Lee DH, Moon MK, et al. Outcomes analysis of surgical and medical treatments for patients with primary aldosteronism. Endocr J. 2017;64(6):623-32.

For peer review only

11. Kline GA, Pasieka JL, Harvey A, So B, Dias VC. Medical or surgical therapy for primary aldosteronism: post-treatment follow-up as a surrogate measure of comparative outcomes. Ann Surg Oncol. 2013;20(7):2274-8.

12. Wachtel H, Fraker DL. Therapeutic Outcomes with Surgical and Medical Management of Primary Aldosteronism. Curr Cardiol Rep. 2021;23(7):89.

13. Sechi LA, Colussi GL, Novello M, Uzzau A, Catena C. Mineralocorticoid Receptor Antagonists and Clinical Outcomes in Primary Aldosteronism: As Good as Surgery? Horm Metab Res. 2015;47(13):1000-6.

14. Chen SY, Chen JY, Huang WC, Puar THK, Chin Kek P, Chueh JS, et al. Cardiovascular outcomes and all-cause mortality in primary aldosteronism after adrenalectomy or mineralocorticoid receptor antagonist treatment: a meta-analysis. Eur J Endocrinol. 2022;187(6):S47-S58.

15. Page MJ, McKenzie JE, Bossuyt PM, Boutron I, Hoffmann TC, Mulrow CD, et al. The PRISMA 2020 statement: an updated guideline for reporting systematic reviews. Bmj. 2021;372:n71.

16. Bramer WM, Giustini D, de Jonge GB, Holland L, Bekhuis T. De-duplication of database search results for systematic reviews in EndNote. J Med Libr Assoc. 2016;104(3):240-3.

17. Ouzzani M, Hammady H, Fedorowicz Z, Elmagarmid A. Rayyan-a web and mobile app for systematic reviews. Syst Rev. 2016;5(1):210.

18. Higgins JP, Thompson SG. Quantifying heterogeneity in a meta-analysis. Stat Med. 2002;21(11):1539-58.

19. Huang WC, Chen YY, Lin YH, Chueh JS. Composite Cardiovascular Outcomes in Patients With Primary Aldosteronism Undergoing Medical Versus Surgical Treatment: A Meta-Analysis. Front Endocrinol (Lausanne). 2021;12:644260.

20. Rayner B. Primary aldosteronism and aldosterone-associated hypertension. J Clin Pathol. 2008;61(7):825-31.

21. Spyroglou A, Handgriff L, Müller L, Schwarzlmüller P, Parasiliti-Caprino M, Fuss CT, et al. The metabolic phenotype of patients with primary aldosteronism: impact of subtype and sex - a multicenter-study of 3566 Caucasian and Asian subjects. Eur J Endocrinol. 2022;187(3):361-72.

For peer review only

22. Morimoto S, Ichihara A. Management of primary aldosteronism and mineralocorticoid receptor-associated hypertension. Hypertens Res. 2020;43(8):744-53.

23. Satoh M, Maruhashi T, Yoshida Y, Shibata H. Systematic review of the clinical outcomes of mineralocorticoid receptor antagonist treatment versus adrenalectomy in patients with primary aldosteronism. Hypertens Res. 2019;42(6):817-24.

24. Qian N, Xu J, Wang Y. Stroke Risks in Primary Aldosteronism with Different Treatments: A Systematic Review and Meta-Analysis. J Cardiovasc Dev Dis. 2022;9(9).

25. Brunt LM. The positive impact of laparoscopic adrenalectomy on complications of adrenal surgery. Surg Endosc. 2002;16(2):252-7.

26. Colvin J, Krishnamurthy V, Jin J, Shin J, Siperstein A, Berber E. A Comparison of Robotic Versus Laparoscopic Adrenalectomy in Patients With Primary Hyperaldosteronism. Surg Laparosc Endosc Percutan Tech. 2017;27(5):391-3.

27. Hundemer GL, Curhan GC, Yozamp N, Wang M, Vaidya A. Cardiometabolic outcomes and mortality in medically treated primary aldosteronism: a retrospective cohort study. Lancet Diabetes Endocrinol. 2018;6(1):51-9.

28. Reincke M, Fischer E, Gerum S, Merkle K, Schulz S, Pallauf A, et al. Observational study mortality in treated primary aldosteronism: the German Conn's registry. Hypertension. 2012;60(3):618-24.

29. Rossi GP, Cesari M, Cuspidi C, Maiolino G, Cicala MV, Bisogni V, et al. Long-term control of arterial hypertension and regression of left ventricular hypertrophy with treatment of primary aldosteronism. Hypertension. 2013;62(1):62-9.

30. Sechi LA, Novello M, Lapenna R, Baroselli S, Nadalini E, Colussi GL, et al. Long-term renal outcomes in patients with primary aldosteronism. JAMA. 2006;295(22):2638-45.

For peer review only

1

PRISMA-P 2015 Checklist

This checklist has been adapted for use with protocol submissions to *Systematic Reviews* from Table 3 in Moher D et al: Preferred reporting items for systematic review and meta-analysis protocols (PRISMA-P) 2015 statement. *Systematic Reviews* 2015 **4**:1

Section/topic	#	Checklist item	Information reported		Line number(s)
			Yes	No	
**ADMINISTRATIVE INFORMATION**					
**Title**					
Identification	1a	Identify the report as a protocol of a systematic review	X		
Update	1b	If the protocol is for an update of a previous systematic review, identify as such		X	
**Registration**	2	If registered, provide the name of the registry (e.g., PROSPERO) and registration number in the Abstract	X		
**Authors**					
Contact	3a	Provide name, institutional affiliation, and e-mail address of all protocol authors; provide physical mailing address of corresponding author	X		
Contributions	3b	Describe contributions of protocol authors and identify the guarantor of the review	X		
**Amendments**	4	If the protocol represents an amendment of a previously completed or published protocol, identify as such and list changes; otherwise, state plan for documenting important protocol amendments		X	
**Support**					
Sources	5a	Indicate sources of financial or other support for the review	X		
Sponsor	5b	Provide name for the review funder and/or sponsor	X		
Role of sponsor/funder	5c	Describe roles of funder(s), sponsor(s), and/or institution(s), if any, in developing the protocol		X	
**INTRODUCTION**					
**Rationale**	6	Describe the rationale for the review in the context of what is already known	X		
**Objectives**	7	Provide an explicit statement of the question(s) the review will address with reference to participants, interventions, comparators, and outcomes (PICO)	X		
**METHODS**					

BioMed Central
The Open Access Publisher

Section/topic	#	Checklist item	Information reported		Line number(s)
			Yes	No	
**Eligibility criteria**	8	Specify the study characteristics (e.g., PICO, study design, setting, time frame) and report characteristics (e.g., years considered, language, publication status) to be used as criteria for eligibility for the review	X		
**Information sources**	9	Describe all intended information sources (e.g., electronic databases, contact with study authors, trial registers, or other grey literature sources) with planned dates of coverage	X		
**Search strategy**	10	Present draft of search strategy to be used for at least one electronic database, including planned limits, such that it could be repeated		X	
STUDY RECORDS					
Data management	11a	Describe the mechanism(s) that will be used to manage records and data throughout the review	X		
Selection process	11b	State the process that will be used for selecting studies (e.g., two independent reviewers) through each phase of the review (i.e., screening, eligibility, and inclusion in meta-analysis)	X		
Data collection process	11c	Describe planned method of extracting data from reports (e.g., piloting forms, done independently, in duplicate), any processes for obtaining and confirming data from investigators	X		
**Data items**	12	List and define all variables for which data will be sought (e.g., PICO items, funding sources), any pre-planned data assumptions and simplifications	X		
**Outcomes and prioritization**	13	List and define all outcomes for which data will be sought, including prioritization of main and additional outcomes, with rationale	X		
**Risk of bias in individual studies**	14	Describe anticipated methods for assessing risk of bias of individual studies, including whether this will be done at the outcome or study level, or both; state how this information will be used in data synthesis	X		
DATA					
**Synthesis**	15a	Describe criteria under which study data will be quantitatively synthesized	X		
	15b	If data are appropriate for quantitative synthesis, describe planned summary measures, methods of handling data, and methods of combining data from studies, including any planned exploration of consistency (e.g., I^2, Kendall's tau)	X		
	15c	Describe any proposed additional analyses (e.g., sensitivity or subgroup analyses, meta-regression)	X		
	15d	If quantitative synthesis is not appropriate, describe the type of summary planned		X	
**Meta-bias(es)**	16	Specify any planned assessment of meta-bias(es) (e.g., publication bias across studies, selective reporting within studies)	X		
**Confidence in cumulative evidence**	17	Describe how the strength of the body of evidence will be assessed (e.g., GRADE)	X		

BioMed Central The Open Access Publisher

BMJ Open

Therapeutic Outcomes with Surgical and Medical Management for Primary Aldosteronism: protocol for a Systematic Review and Meta-Analysis

Journal:	*BMJ Open*
Manuscript ID	bmjopen-2023-072585.R2
Article Type:	Protocol
Date Submitted by the Author:	13-Jul-2023
Complete List of Authors:	Rocca, Aldo; University of Molise, Department of Medicine and Health Science “V. Tiberio”<br>Gkaniatsa, Eleftheria; University of Gothenburg, Department of Internal Medicine and Clinical Nutrition, Institute of Medicine, Sahlgrenska Academy,; Sahlgrenska University Hospital, Department of Endocrinology<br>Brunese, Maria Chiara; University of Molise, Department of Medicine and Health Science “V. Tiberio”<br>Hessman, Eva; Gothenburg University Library, Biomedical Library,<br>Muth, Andreas; Sahlgrenska University Hospital, Department of Surgery, ; University of Gothenburg, Department of Surgery, Institute of Clinical Sciences, Sahlgrenska Academy<br>Nwaru, Bright; University of Gothenburg, Krefting Research Centre; University of Gothenburg, Wallenberg Centre for Molecular and Translational Medicine, Institute of Medicine,<br>Ragnarsson, Oskar; Sahlgrenska University Hospital, Department of Endocrinology; University of Gothenburg, Department of Internal Medicine and Clinical Nutrition, Institute of Medicine, Sahlgrenska Academy<br>Bobbio, Emanuele; Sahlgrenska University Hospital, Department of Cardiology; University of Gothenburg, Institute of Medicine at Sahlgrenska Academy<br>Esposito, Daniela; University of Gothenburg, Department of Internal Medicine and Clinical Nutrition, Institute of Medicine, Sahlgrenska Academy, ; Sahlgrenska University Hospital, Department of Endocrinology
**Primary Subject Heading**:	Diabetes and endocrinology
Secondary Subject Heading:	Cardiovascular medicine
Keywords:	Hypertension < CARDIOLOGY, General endocrinology < DIABETES & ENDOCRINOLOGY, Endocrine tumours < DIABETES & ENDOCRINOLOGY, Adrenal disorders < DIABETES & ENDOCRINOLOGY

Therapeutic Outcomes with Surgical and Medical Management for Primary Aldosteronism: protocol for a Systematic Review and Meta-Analysis

Aldo Rocca[1], MD, Eleftheria Gkaniatsa[2,3], MD, Maria Chiara Brunese[1], MD, Eva Hessman[4], Andreas Muth[5,6], MD, PhD, Bright I. Nwaru[7,8], PhD, Oskar Ragnarsson[2,3,8], MD, PhD, Emanuele Bobbio[9,10]*, MD, Daniela Esposito[2,3]*, MD, PhD

*these authors contributed equally

[1] Department of Medicine and Health Science "V. Tiberio", University of Molise, Campobasso, Italy
[2] Department of Internal Medicine and Clinical Nutrition, Institute of Medicine, Sahlgrenska Academy, University of Gothenburg, Gothenburg, Sweden
[3] Department of Endocrinology, Sahlgrenska University Hospital, Gothenburg, Sweden
[4] Biomedical Library, Gothenburg University Library, University of Gothenburg, Gothenburg, Sweden
[5] Department of Surgery, Sahlgrenska University Hospital, Gothenburg, Sweden
[6] Department of Surgery, Institute of Clinical Sciences, Sahlgrenska Academy, University of Gothenburg, Gothenburg, Sweden
[7] Krefting Research Centre, Institute of Medicine, Sahlgrenska Academy, University of Gothenburg, Gothenburg, Sweden
[8] Wallenberg Centre for Molecular and Translational Medicine, Institute of Medicine, University of Gothenburg, Gothenburg, Sweden
[9] Department of Cardiology, Sahlgrenska University Hospital, Gothenburg, Sweden
[10] Institute of Medicine at Sahlgrenska Academy, University of Gothenburg, Gothenburg, Sweden

Key Words: hyperaldosteronism, adrenalectomy, MR antagonists, spironolactone, eplerenone, meta-analysis

Abstract: 257 words

Manuscript: 1992 words

References: 30

Tables and figures: 1

Supplementary material: 1

For peer review only

Running title: Therapeutic outcomes in primary aldosteronism

Corresponding author and person to whom reprint requests should be addressed:

Daniela Esposito, MD, PhD, Department of Endocrinology, Sahlgrenska University Hospital Institute of Medicine, Sahlgrenska Academy, University of Gothenburg, Gothenburg, Sweden.

Email: daniela.esposito@gu.se

For peer review only

E-mail address of all protocol authors:

Aldo Rocca: aldo.rocca@unimol.it

Eleftheria Gkaniatsa: eleftheria.gkaniatsa@vgregion.se

Maria Chiara Brunese: mariachiarabrunese@gmail.com

Eva Hessman: eva.hessman@ub.gu.se

Andreas Muth: andreas.muth@vgregion.se

Bright I. Nwaru: bright.nwaru@gu.se

Oskar Ragnarsson: oskar.ragnarsson@medic.gu.se

Emanuele Bobbio: emanuele.bobbio@vgregion.se

Daniela Esposito: daniela.esposito@gu.se

For peer review only

Abstract

Introduction: Treatment strategies for primary aldosteronism (PA) include unilateral adrenalectomy and medical treatment with mineralocorticoid receptor (MR) antagonists. Whether these two different treatment strategies are comparable in mitigating the detrimental effect of PA on outcomes is still debated.

Objectives: The primary aim of this systematic review is to identify, appraise, and synthesize existing literature comparing clinical outcomes after treatment in patients with PA.

Methods and analysis: A systematic and comprehensive search will be performed using PubMed, Web of Science, and EMBASE, for studies published until December 2022. Observational and interventional studies will be eligible for inclusion. The quality of observational studies will be assessed using the Newcastle–Ottawa scale, while interventional studies will be assessed using the Cochrane Effective Practice Organization of Care tool. The collected evidence will be narratively synthesized. We will perform meta-analysis to pool estimates from studies considered to be homogenous. Reporting of the systematic review and meta-analysis will be in accordance to the Meta-analysis of Observational Studies in Epidemiology Preferred Reporting Items for Systematic reviews and Meta-Analysis guidelines.

Ethics and dissemination: As this study is based solely on the published literature, no ethics approval is required. This review will aim to provide some estimates on outcomes, including survival, rates of clinical and biochemical control, cardiovascular and cerebrovascular events, as well as data on quality of life and renal function, in patients with PA treated surgically or with MR antagonists. The study findings will be presented at scientific meetings and will be published in an international peer-reviewed scientific journal.

PROSPERO registration number: CRD42022362506.

For peer review only

Strengths and limitations of this study

- Findings from this systematic review will provide a comprehensive and most contemporaneous synthesis of the underlying evidence regarding long-term outcomes in patients with primary aldosteronism (PA) who have been treated with either surgery or mineralocorticoid receptor (MR) antagonists.
- This systematic review protocol follows the Preferred Reporting Items for Systematic Review and Meta-Analysis Protocols guidelines.
- The identification of studies from leading medical and public health databases, with no geographical or language limitations, will advance the import of this evidence synthesis across settings.
- The reproducibility of our work is enhanced through a priori outline of the review processes before the actual review starts.

For peer review only

For peer review only - http://bmjopen.bmj.com/site/about/guidelines.xhtml

1. Background

Primary aldosteronism (PA), also known as Conn´s syndrome, is the most common cause of secondary hypertension, affecting 3–13% of hypertensive patients and 20% of patients with therapy-resistant hypertension (1). PA is characterized by autonomous hypersecretion of aldosterone, causing inappropriate potassium excretion, concurrent sodium and fluid retention, as well as low plasma renin concentration (1, 2). PA is most commonly caused by adrenal adenoma or unilateral or bilateral adrenal hyperplasia (2). Rare causes of PA include adrenocortical carcinoma and inherited forms of familial hyperaldosteronism (1, 2). Patients with PA are at higher risk of cardiovascular diseases such as myocardial infarction, stroke and atrial fibrillation than patients with essential hypertension also when adjusted for blood pressure (BP) (3, 4).

Treatment strategies for PA include unilateral adrenalectomy and medical treatment with mineralocorticoid receptor (MR) antagonists (1). Minimally invasive adrenalectomy is a safe and feasible procedure and recommended treatment for patients with unilateral PA (5-7). Although adrenalectomy usually results in biochemical cure, hypertension can persist in up to 60% of patients (7). Conversely, MR antagonists (spironolactone or eplerenone) are an option in patients with bilateral PA or when surgery is not feasible (8). Furthermore, medical treatment is preferred in case of uncertain lateralization, especially at centers where adrenal venous sampling is not available or has been unsuccessful (9).

Evidence is still limited on whether different treatment strategies mitigate the detrimental effect of PA on short- and long-term outcomes (10). Some investigators reported similar outcomes between surgical and medical treatment (11-13). However, other studies showed better control of arterial BP and hypokalemia, better cardiovascular outcomes, as well as improved quality of life after adrenalectomy than with medical treatment (5). In a recent meta-analysis, lower risk of all-cause mortality and/or major adverse cardiovascular events (MACE) were also reported in surgically treated patients compared with those treated with MR antagonists (14). However, evidence about the effect of adrenalectomy *vs* MR antagonists on clinical and/or biochemical control as well as quality of life and renal function is still debated. Furthermore, evaluation of effects and side effects of MR antagonist treatment is hampered by patients frequently being undertreated. Thus, it is paramount to identify and synthesize the available evidence to date in order to gain clearer insights into this field.

For peer review only

2. Scientific questions and objectives

The aim of this systematic review is to identify, critically appraise, and synthesize available data evaluating whether patients with PA who underwent adrenalectomy have higher survival rate than patients treated with MR antagonists.

The secondary aim of this review is to compare rates of cardiovascular and cerebrovascular events (heart failure, myocardial infarction, stroke), successful clinical control (normalized or improved blood pressure, tapering of antihypertensive medications), biochemical control (normalized plasma renin and potassium levels), as well as improved quality of life and renal function between patients who underwent adrenalectomy and those treated with MR antagonists.

For peer review only

3. Project description

3.1 Methods

We will perform a systematic review and meta-analysis according to Preferred Reporting Items for Systematic Reviews and Meta-Analysis (PRISMA) guidelines (15).

The main study objectives have been formulated according to PICO (Patients, Intervention, Comparison, Outcomes) model (**Table 1**). The protocol for this review has been reported according PRISMA-P guidelines and has been registered in PROSPERO (registration number: CRD42022362506).

This systematic review was planned in September 2022 and is expected to be finalized in January 2024.

Table 1. PICO

**P**	Patients with PA
**I**	Adrenalectomy
**C**	Medical treatment
**O**	*All-cause mortality*<br>*Cardiovascular and cerebrovascular events:* heart failure, myocardial infarction, stroke<br>*Clinical control:* normalized or improved blood pressure, less antihypertensive medication<br>*Biochemical control:* normalized renin and potassium<br>*Quality of life*<br>*Renal function*

3.2 Eligibility criteria and information sources

The population of interest will be patients with PA treated with adrenalectomy in comparison with those receiving MR antagonists. Studies that report survival rates and rates of clinical control (i.e., normalized or improved blood pressure, tapering of antihypertensive medication), biochemical control (i.e. normalized renin and potassium), cardiovascular and cerebrovascular events (heart failure, myocardial infarction, stroke), as well as data on quality of life or renal function will be included.

Data across different age groups, gender, and ethnicity will be analyzed, and there will be no exclusion by country or language. We will translate studies reported in other languages than English.

Observational studies (cross-sectional, cohort, and case–control) and interventional (randomized controlled trials, field trials, community trials, etc.) studies will be included.

Exclusion criteria will be: date of publication before 2000, review articles, case reports, editorials, letters, and other articles where the calculation of rates of outcomes is not feasible.

For peer review only

If an eligible article does not report the rates of the pre-specified outcomes, an attempt to retrieve this data will be performed by contacting the study authors.

Search will be conducted in three databases (PubMed, Web of Science, and EMBASE) using commonly used terms stated in related literature as well as Medical Subject Headings (MeSH) terms. Search strategies will be focused on three concepts: the population/diagnosis, surgical treatment and medical therapy. The following terms will be used: hyperaldosteronism, hyperaldosteronaemia, aldosteronism, aldosteronaemia, adrenalectomy(-ies), surgery, surgical resection, MR antagonists, spironolactone, eplerenone. All databases will be searched from January 2000 until December 2022. The full search strategy for all databases is shown in Supplementary material.

An additional manual-search based on the citations and references for included reports will also be conducted. All reports obtained from the databases will be exported to the reference management software Endnote. The search result will then be deduplicated, following the Bramer method (16).

3.3 Ethical issues

Ethical approval or informed consent are not required since this systematic review will be based only on previously published studies and does not imply any direct contact with individual subjects.

3.4 Selection process

Article screening will be independently performed by authors AR, EG, MB and DE by evaluating titles and abstracts for eligibility assessment. Relevant reports will be identified and analyzed by at least two authors in a blinded process using the web application Rayyan (17). In case of disagreement, a third author will resolve it. Bibliographies and citations for the eligible articles will be examined to identify any additional relevant report, which were not retrieved in the literature search.

The selected reports will be categorized into three groups: irrelevant, relevant, and unsure. The reports categorized as irrelevant by both reviewers will be excluded. The reports categorized as relevant or unsure by at least one reviewer will be retrieved and the full papers evaluated. Then, for all the potentially eligible reports, the full texts will be carefully reviewed and a selection of reports to include will be made by each reviewer. The reports selected by

For peer review only

For peer review only - http://bmjopen.bmj.com/site/about/guidelines.xhtml

the reviewers will be compared and any discrepancies will be discussed. When an agreement is not reached, another author (OR) will make the final decision.

The search and selection process will be presented in a PRISMA flowchart.

3.5 Data extraction

Relevant information from included studies will be extracted using a data extraction form that will help to standardize the data extraction process. The following data will be collected: study name, name of the first author, year of publication, country where the study was conducted, total number of patients, source from which study subjects were selected, study design, aetiology of PA, definition of diagnostic criteria for PA, outcomes definition, gender, age, ethnicity, incidence with 95% confidence intervals (CIs) of the outcomes. When possible, missing information will be calculated from the available data. Authors will be contacted by the reviewers if data are inadequate, unclear, or missing from the report. At least two reviewers will independently extract data from all studies. Any discrepancies between reviewers in the data extraction will be resolved by either consensus or a third reviewer will arbitrate.

3.6 Quality assessment

Quality of observational studies will be assessed using the Newcastle – Ottawa Scale (NOS), while the Cochrane Effective Practice Organization of Care tool will be used for interventional studies. Two reviewers will perform the quality appraisal and a third reviewer will arbitrate any disagreement.

3.7 Data synthesis

If possible, relative Risk (RR) estimates together with Confidence Intervals (CI) will be calculated from each study and, if the studies are sufficiently homogeneous, a meta-analysis will be performed by pooling estimates across studies using the random effects approach. Heterogeneity will be examined using the Higgins I^2 statistic (18). Heterogeneity will be considered minimal if I^2 values will be between 0-30%, moderate if I^2 will be between 31-50%, and high if I^2 >50%.

For peer review only - http://bmjopen.bmj.com/site/about/guidelines.xhtml

Publication bias will be assessed using Begg's funnel plot and Egger's test. An asymmetrical funnel plot or P value < 0.10 on Egger's test will be considered as an indication of publication bias. If data are sufficient, subgroup analyses will be performed based on the quality of study, age, gender, etiology of PA, disease severity, diagnostic criteria for PA, and ethnicity.

3.8 Patient and Public Involvement

None

4. Discussion

Once thought a rare disease, PA is nowadays known to be the most frequent form of secondary hypertension, affecting 3–13% of hypertensive patients (1, 19). PA is associated with higher risk of metabolic, renal, and cardiovascular complications compared with essential hypertension (20). The current treatment options for PA includes surgical adrenalectomy or medical therapy with MR antagonists and the choice of treatment is based on PA subtype classifications and patient preference (14, 21, 22). In small clinical studies, surgical treatment has been shown to have better outcomes (9, 10, 19). However, large, randomized trials are lacking, and no solid conclusion can be drawn.

Previous systematic reviews and meta-analyses performed on this topic (5, 14, 23, 24) have mainly assessed the impact of adrenalectomy vs medical treatment on all-cause mortality and major adverse cardiovascular events (*e.g.* coronary artery disease, stroke, arrhythmia, and heart failure) in PA patients. However, other treatment outcomes, as clinical and biochemical control, quality of life and other comorbidities (rather than cardiovascular) have not been assessed yet.

The comparison between surgical and medical treatment in PA is challenging. Surgical approach has changed over time, with introduction of minimally invasive surgery and robotic approaches that have modified the panorama of the PA treatment, allowing for organ-sparing resection and improved surgical outcomes (25, 26). Therefore, studies published before 2000 will be excluded in the present meta-analysis. Another possible pitfall could be that patients receiving MR antagonists are often undertreated, resulting in an inadequate biochemical and clinical control of the disease (6, 27). In undertreated patients, the plasma renin remains

For peer review only - http://bmjopen.bmj.com/site/about/guidelines.xhtml

suppressed, which *per se* results in increased risk of cardiovascular events, renal dysfunction, and mortality (6). On the other hand, adrenalectomy solves the root cause of PA and, thus, the MR over-activation, making the comparison with medical treatment challenging (6). It is also important to consider that patients who are treated with surgery may have different disease characteristics compared to those treated medically (*e.g.* unilateral vs. bilateral PA or uncertain lateralization), which may affect treatment outcomes. Additionally, many PA patients may not be suitable candidates for surgery due to various reasons, such as patient preference or comorbidities. These factors can confound the comparison between surgical and medical therapy for PA; therefore the interpretation of the results of this comparison should be done with caution, and individualized treatment decisions should be made (28-30).

Still, no previous study has synthesized the available evidence to date about the effect of different treatment strategies on clinical and/or biochemical control as well as quality of life and renal function. In the present review, we will investigate long-term outcomes in patients with PA who have been treated with either surgery or MR antagonists. In addition, we will gather information on demographic characteristics (age, gender, and ethnicity/race) in order to explore their potential impact on treatment outcomes. Overall, we believe that our systemic review will provide valuable information for optimizing treatment strategies in patients with PA.

Ethics and dissemination

As this study is based solely on the published literature, no ethics approval is required. The study findings will be presented at scientific meetings and will be published in an international peer-reviewed scientific journal.

Acknowledgements

We thank medical librarians Mrs. Eva Hessman and Mrs. Linda Hammarbäck at the Biomedical Library, Gothenburg University Library, University of Gothenburg, Gothenburg, Sweden for performing the systematical search in the literature and for their valuable feedback on our search strategy and methodology.

For peer review only

Contributions

OR and DE conceived this study. AR, EG, MCB, EB and DE developed the study protocol and will implement the systematic review under the supervision of AM, BN, and OR. EB and BN will provide the statistical analysis expertise. EB, BN, and DE will conduct data analysis. EH will develop the search strings and conduct the study search. AR, EG, MCB, and DE will take part in the study search, and will perform the screening and extraction of data whereas AM, BN, OR, and EB will review the work. DE wrote the first manuscript draft and all authors gave input to the final draft of the protocol.

Funding statement

This work received support from the Swedish state under the agreement between the Swedish government and the county councils, the ALF-agreement (ALFGBG-874631).

Conflicts of interest and financial disclosure

None declared

For peer review only

References

1. Young WF. Primary aldosteronism: renaissance of a syndrome. Clin Endocrinol (Oxf). 2007;66(5):607-18.

2. Conn JW, Louis LH. Primary aldosteronism: a new clinical entity. Trans Assoc Am Physicians. 1955;68:215-31; discussion, 31-3.

3. Monticone S, D'Ascenzo F, Moretti C, Williams TA, Veglio F, Gaita F, et al. Cardiovascular events and target organ damage in primary aldosteronism compared with essential hypertension: a systematic review and meta-analysis. Lancet Diabetes Endocrinol. 2018;6(1):41-50.

4. Catena C, Colussi G, Nadalini E, Chiuch A, Baroselli S, Lapenna R, et al. Cardiovascular outcomes in patients with primary aldosteronism after treatment. Arch Intern Med. 2008;168(1):80-5.

5. Muth A, Ragnarsson O, Johannsson G, Wangberg B. Systematic review of surgery and outcomes in patients with primary aldosteronism. Br J Surg. 2015;102(4):307-17.

6. Funder JW, Carey RM, Mantero F, Murad MH, Reincke M, Shibata H, et al. The Management of Primary Aldosteronism: Case Detection, Diagnosis, and Treatment: An Endocrine Society Clinical Practice Guideline. J Clin Endocrinol Metab. 2016;101(5):1889-916.

7. Iacobone M, Citton M, Viel G, Rossi GP, Nitti D. Approach to the surgical management of primary aldosteronism. Gland Surg. 2015;4(1):69-81.

8. Stavropoulos K, Papadopoulos C, Koutsampasopoulos K, Lales G, Mitas C, Doumas M. Mineralocorticoid Receptor Antagonists in Primary Aldosteronism. Curr Pharm Des. 2018;24(46):5508-16.

9. Williams TA, Burrello J, Sechi LA, Fardella CE, Matrozova J, Adolf C, et al. Computed Tomography and Adrenal Venous Sampling in the Diagnosis of Unilateral Primary Aldosteronism. Hypertension. 2018;72(3):641-9.

10. Park KS, Kim JH, Yang YS, Hong AR, Lee DH, Moon MK, et al. Outcomes analysis of surgical and medical treatments for patients with primary aldosteronism. Endocr J. 2017;64(6):623-32.

For peer review only

11. Kline GA, Pasieka JL, Harvey A, So B, Dias VC. Medical or surgical therapy for primary aldosteronism: post-treatment follow-up as a surrogate measure of comparative outcomes. Ann Surg Oncol. 2013;20(7):2274-8.

12. Wachtel H, Fraker DL. Therapeutic Outcomes with Surgical and Medical Management of Primary Aldosteronism. Curr Cardiol Rep. 2021;23(7):89.

13. Sechi LA, Colussi GL, Novello M, Uzzau A, Catena C. Mineralocorticoid Receptor Antagonists and Clinical Outcomes in Primary Aldosteronism: As Good as Surgery? Horm Metab Res. 2015;47(13):1000-6.

14. Chen SY, Chen JY, Huang WC, Puar THK, Chin Kek P, Chueh JS, et al. Cardiovascular outcomes and all-cause mortality in primary aldosteronism after adrenalectomy or mineralocorticoid receptor antagonist treatment: a meta-analysis. Eur J Endocrinol. 2022;187(6):S47-S58.

15. Page MJ, McKenzie JE, Bossuyt PM, Boutron I, Hoffmann TC, Mulrow CD, et al. The PRISMA 2020 statement: an updated guideline for reporting systematic reviews. Bmj. 2021;372:n71.

16. Bramer WM, Giustini D, de Jonge GB, Holland L, Bekhuis T. De-duplication of database search results for systematic reviews in EndNote. J Med Libr Assoc. 2016;104(3):240-3.

17. Ouzzani M, Hammady H, Fedorowicz Z, Elmagarmid A. Rayyan-a web and mobile app for systematic reviews. Syst Rev. 2016;5(1):210.

18. Higgins JP, Thompson SG. Quantifying heterogeneity in a meta-analysis. Stat Med. 2002;21(11):1539-58.

19. Huang WC, Chen YY, Lin YH, Chueh JS. Composite Cardiovascular Outcomes in Patients With Primary Aldosteronism Undergoing Medical Versus Surgical Treatment: A Meta-Analysis. Front Endocrinol (Lausanne). 2021;12:644260.

20. Rayner B. Primary aldosteronism and aldosterone-associated hypertension. J Clin Pathol. 2008;61(7):825-31.

21. Spyroglou A, Handgriff L, Müller L, Schwarzlmüller P, Parasiliti-Caprino M, Fuss CT, et al. The metabolic phenotype of patients with primary aldosteronism: impact of subtype and sex - a multicenter-study of 3566 Caucasian and Asian subjects. Eur J Endocrinol. 2022;187(3):361-72.

For peer review only

For peer review only - http://bmjopen.bmj.com/site/about/guidelines.xhtml

22. Morimoto S, Ichihara A. Management of primary aldosteronism and mineralocorticoid receptor-associated hypertension. Hypertens Res. 2020;43(8):744-53.

23. Satoh M, Maruhashi T, Yoshida Y, Shibata H. Systematic review of the clinical outcomes of mineralocorticoid receptor antagonist treatment versus adrenalectomy in patients with primary aldosteronism. Hypertens Res. 2019;42(6):817-24.

24. Qian N, Xu J, Wang Y. Stroke Risks in Primary Aldosteronism with Different Treatments: A Systematic Review and Meta-Analysis. J Cardiovasc Dev Dis. 2022;9(9).

25. Brunt LM. The positive impact of laparoscopic adrenalectomy on complications of adrenal surgery. Surg Endosc. 2002;16(2):252-7.

26. Colvin J, Krishnamurthy V, Jin J, Shin J, Siperstein A, Berber E. A Comparison of Robotic Versus Laparoscopic Adrenalectomy in Patients With Primary Hyperaldosteronism. Surg Laparosc Endosc Percutan Tech. 2017;27(5):391-3.

27. Hundemer GL, Curhan GC, Yozamp N, Wang M, Vaidya A. Cardiometabolic outcomes and mortality in medically treated primary aldosteronism: a retrospective cohort study. Lancet Diabetes Endocrinol. 2018;6(1):51-9.

28. Reincke M, Fischer E, Gerum S, Merkle K, Schulz S, Pallauf A, et al. Observational study mortality in treated primary aldosteronism: the German Conn's registry. Hypertension. 2012;60(3):618-24.

29. Rossi GP, Cesari M, Cuspidi C, Maiolino G, Cicala MV, Bisogni V, et al. Long-term control of arterial hypertension and regression of left ventricular hypertrophy with treatment of primary aldosteronism. Hypertension. 2013;62(1):62-9.

30. Sechi LA, Novello M, Lapenna R, Baroselli S, Nadalini E, Colussi GL, et al. Long-term renal outcomes in patients with primary aldosteronism. JAMA. 2006;295(22):2638-45.

For peer review only

Supplementary material. Full search strategies used in PubMed, Web of Science, and EMBASE to identify relevant articles for the systematic review.

PubMed

#	Searches
#1	hyperaldosteronism[tiab] OR hyperaldosteronemia[tiab] OR aldosteronism[tiab] OR aldosteronemia[tiab] OR Hyperaldosteronism[mh] OR Conn Syndrome[tiab] OR Primary Hyperaldosteronism[tiab] OR Conn's Syndrome[tiab] OR Conns Syndrome[tiab] OR Syndrome, Conn*[tiab]
#2	Adrenalectomy[mh] OR adrenalectomy[tiab] OR adrenalectomies[tiab] OR surgery[tiab] OR surgical[tiab] OR resection[tiab] OR management[tiab] OR treatment[tiab] OR drug therapy[mh] OR medical therapy[tiab] OR MR antagonists[tiab] OR MR antagonist[tiab] OR mineralocorticoid receptor antagonists[tiab] OR mineralocorticoid receptor antagonist[tiab] OR Spironolactone[mh] OR spironolactone[tiab] OR eplerenone[mh] OR eplerenone[tiab] OR Spirolactone[tiab] OR Veroshpiron[tiab] OR Verospirone[tiab] OR Spiractin[tiab] OR Spirogamma[tiab] OR Spirolang[tiab] OR Aldactone[tiab] OR Verospiron[tiab] OR Aldactone A[tiab] OR Aquareduct[tiab] OR Espironolactona Alter[tiab] OR SC-9420[tiab] OR SC 9420[tiab] OR SC9420[tiab] OR Inspra[tiab]
#3	#1 AND #2
#4	Animals[mh] NOT humans[mh]
#5	#3 NOT #4
#6	Limit to year 2000-

For peer review only

Web of Science Core Collection

#	Searches
#1	TS=(hyperaldosteronism OR hyperaldosteronemia OR aldosteronism OR aldosteronemia OR Hyperaldosteronism OR "Conn Syndrome" OR "Primary Hyperaldosteronism" OR "Conn's Syndrome" OR "Conns Syndrome" OR "Syndrome, Conn*")
#2	TS=(Adrenalectomy OR adrenalectomies OR surgery OR surgical OR resection OR management OR treatment OR "drug therapy" OR "medical therapy" OR ”MR antagonist” OR "MR antagonists" OR ”mineralocorticoid receptor antagonists” OR ”mineralocorticoid receptor antagonist” OR Spironolactone OR spironolactone OR eplerenone OR eplerenone OR Spirolactone OR Veroshpiron OR Verospirone OR Spiractin OR Spirogamma OR Spirolang OR Aldactone OR Verospiron OR "Aldactone A" OR Aquareduct OR "Espironolactona Alter" OR SC-9420 OR "SC 9420" OR SC9420 OR Inspra)
#3	#1 AND #2
#4	ALL=human*
#5	#3 NOT #4
#6	Limit to year 2000-, Articles

For peer review only

Embase

#	Searches
#1	hyperaldosteronism:ti,ab OR hyperaldosteronemia:ti,ab OR aldosteronism:ti,ab OR aldosteronemia:ti,ab OR Hyperaldosteronism/exp OR 'Conn Syndrome':ti,ab OR 'Primary Hyperaldosteronism':ti,ab OR 'Conns Syndrome':ti,ab OR 'Syndrome, Conn*':ti,ab
#2	Adrenalectomy/exp OR adrenalectomy:ti,ab OR adrenalectomies:ti,ab OR surgery/exp OR surgery:ti,ab OR surgical:ti,ab OR resection:ti,ab OR mangament/exp OR management:ti,ab OR treatment:ti,ab OR 'drug therapy'/exp OR 'medical therapy':ti,ab OR 'MR antagonists':ti,ab OR ’MR antagonist’:ti,ab OR ’mineralocorticoid receptor antagonists’:ti,ab OR ’mineralocorticoid receptor antagonist’:ti,ab OR Spironolactone/exp OR spironolactone:ti,ab OR eplerenone/exp OR eplerenone:ti,ab OR Spirolactone:ti,ab OR Veroshpiron:ti,ab OR Verospirone:ti,ab OR Spiractin:ti,ab OR Spirogamma:ti,ab OR Spirolang:ti,ab OR Aldactone:ti,ab OR Verospiron:ti,ab OR 'Aldactone A':ti,ab OR Aquareduct:ti,ab OR 'Espironolactona Alter':ti,ab OR SC-9420:ti,ab OR 'SC 9420':ti,ab OR SC9420:ti,ab OR Inspra:ti,ab
#3	#1 AND #2
#4	('animal'/exp OR animal:ti,ab) NOT (human/exp OR human:ti,ab)
#5	#3 NOT #4
#6	Limit to year 2000-, Articles

For peer review only

PRISMA-P 2015 Checklist

This checklist has been adapted for use with protocol submissions to *Systematic Reviews* from Table 3 in Moher D et al: Preferred reporting items for systematic review and meta-analysis protocols (PRISMA-P) 2015 statement. *Systematic Reviews* 2015 **4**:1

Section/topic	#	Checklist item	Information reported Yes	Information reported No	Line number(s)
**ADMINISTRATIVE INFORMATION**					
**Title**					
Identification	1a	Identify the report as a protocol of a systematic review	X		
Update	1b	If the protocol is for an update of a previous systematic review, identify as such		X	
**Registration**	2	If registered, provide the name of the registry (e.g., PROSPERO) and registration number in the Abstract	X		
**Authors**					
Contact	3a	Provide name, institutional affiliation, and e-mail address of all protocol authors; provide physical mailing address of corresponding author	X		
Contributions	3b	Describe contributions of protocol authors and identify the guarantor of the review	X		
**Amendments**	4	If the protocol represents an amendment of a previously completed or published protocol, identify as such and list changes; otherwise, state plan for documenting important protocol amendments		X	
**Support**					
Sources	5a	Indicate sources of financial or other support for the review	X		
Sponsor	5b	Provide name for the review funder and/or sponsor	X		
Role of sponsor/funder	5c	Describe roles of funder(s), sponsor(s), and/or institution(s), if any, in developing the protocol		X	
**INTRODUCTION**					
**Rationale**	6	Describe the rationale for the review in the context of what is already known	X		
**Objectives**	7	Provide an explicit statement of the question(s) the review will address with reference to participants, interventions, comparators, and outcomes (PICO)	X		
**METHODS**					

For peer review only

BioMed Central The Open Access Publisher

Section/topic	#	Checklist item	Information reported Yes	Information reported No	Line number(s)
**Eligibility criteria**	8	Specify the study characteristics (e.g., PICO, study design, setting, time frame) and report characteristics (e.g., years considered, language, publication status) to be used as criteria for eligibility for the review	X		
**Information sources**	9	Describe all intended information sources (e.g., electronic databases, contact with study authors, trial registers, or other grey literature sources) with planned dates of coverage	X		
**Search strategy**	10	Present draft of search strategy to be used for at least one electronic database, including planned limits, such that it could be repeated		X	
STUDY RECORDS					
Data management	11a	Describe the mechanism(s) that will be used to manage records and data throughout the review	X		
Selection process	11b	State the process that will be used for selecting studies (e.g., two independent reviewers) through each phase of the review (i.e., screening, eligibility, and inclusion in meta-analysis)	X		
Data collection process	11c	Describe planned method of extracting data from reports (e.g., piloting forms, done independently, in duplicate), any processes for obtaining and confirming data from investigators	X		
**Data items**	12	List and define all variables for which data will be sought (e.g., PICO items, funding sources), any pre-planned data assumptions and simplifications	X		
**Outcomes and prioritization**	13	List and define all outcomes for which data will be sought, including prioritization of main and additional outcomes, with rationale	X		
**Risk of bias in individual studies**	14	Describe anticipated methods for assessing risk of bias of individual studies, including whether this will be done at the outcome or study level, or both; state how this information will be used in data synthesis	X		
DATA					
**Synthesis**	15a	Describe criteria under which study data will be quantitatively synthesized	X		
	15b	If data are appropriate for quantitative synthesis, describe planned summary measures, methods of handling data, and methods of combining data from studies, including any planned exploration of consistency (e.g., I^2, Kendall's tau)	X		
	15c	Describe any proposed additional analyses (e.g., sensitivity or subgroup analyses, meta-regression)	X		
	15d	If quantitative synthesis is not appropriate, describe the type of summary planned		X	
**Meta-bias(es)**	16	Specify any planned assessment of meta-bias(es) (e.g., publication bias across studies, selective reporting within studies)	X		
**Confidence in cumulative evidence**	17	Describe how the strength of the body of evidence will be assessed (e.g., GRADE)	X		

For peer review only

BioMed Central The Open Access Publisher
